# Comparison between Ogawa-Kudoh and modified Petroff techniques for mycobacteria cultivation in the diagnosis of pulmonary tuberculosis

**DOI:** 10.1590/S1679-45082018AO4214

**Published:** 2018-06-05

**Authors:** Ronaldo Rodrigues da Costa, Suzane Fernandes da Silva, Romário Costa Fochat, Raquel Leite Macedo, Thamiris Vilela Pereira, Marcio Roberto Silva, Carmen Perches Gomide Pinto, Isabel Cristina Gonçalves Leite

**Affiliations:** 1 Hospital Regional João Penido, Fundação Hospitalar do Estado de Minas Gerais,Juiz de Fora, MG, Brazil.; 2 Hospital Universitário, Universidade Federal de Juiz de Fora, Juiz de Fora, MG, Brazil.; 3 Empresa Brasileira de Pesquisa Agropecuária, Embrapa Gado de Leite, Juiz de Fora, MG, Brazil.; 4 Faculdade de Medicina, Universidade Federal de Juiz de Fora, Juiz de Fora, MG, Brazil.

**Keywords:** Ogawa-Kudoh, Petroff, Tuberculosis, pulmonary/diagnosis, Mycobacterium tuberculosis, Sputum, Culture, Ogawa-Kudoh, Petroff, Tuberculose pulmonar/diagnóstico, Mycobacterium tuberculosis, Escarro, Cultura

## Abstract

**Objective:**

To compare the performance of the Ogawa-Kudoh method with the modified Petroff technique in diagnosis of pulmonary tuberculosis.

**Methods:**

A total of 205 sputum samples from 166 patients with clinical suspicion or under pulmonary tuberculosis follow-up, seen at a public tertiary care hospital, from July 2014 to July 2016 were used. All samples were simultaneously processed using the Ogawa-Kudoh and modified Petroff decontamination methods, according to the recommendations of the Ministry of Health. In the statistical analysis, the McNemar test and the Kappa index were used, respectively, to compare proportions and verify agreement between data.

**Results:**

The Ogawa-Kudoh and modified Petroff methods were efficient in mycobacteria detection, with no significant differences in results (p=0.549) and contamination rate of the cultures (p=0.065). The agreement between techniques was considered excellent (Kappa index of 0.877) and Ogawa-Kudoh, as compared to the modified Petroff technique, showed sensitivity of 90.4%, specificity of 96.6%, positive predictive value of 94.3% and negative predictive value of 94.2%.

**Conclusion:**

The Ogawa-Kudoh technique proved to be sufficiently sensitive and specific for diagnosis of pulmonary tuberculosis, and, therefore, suitable for routine laboratory application. Since it is simple, low-cost and has less technical requirements for biosafety and professional training, Ogawa-Kudoh is an alternative for managers and healthcare professionals to promote the expansion of bacteriological diagnostic coverage of pulmonary tuberculosis.

## RESUMO

**Objetivo:**

Comparar o desempenho do método de Ogawa-Kudoh ao de Petroff modificado no diagnóstico da tuberculose pulmonar.

**Métodos:**

Utilizaram-se 205 amostras de escarro de 166 pacientes com suspeita clínica ou controle de tuberculose pulmonar atendidos em um hospital público terciário, entre os meses de julho de 2014 a julho de 2016. Todas as amostras foram processadas simultaneamente pelos métodos de descontaminação Ogawa-Kudoh e Petroff modificado, seguindo as recomendações do Ministério da Saúde. Na análise estatística, foi empregado o teste de McNemar, para comparação de proporções, e o índice Kappa, para verificar o grau de concordância entre os dados.

**Resultados:**

Os métodos Ogawa-Kudoh e Petroff modificado mostraram-se eficientes na detecção de micobactérias, não sendo verificadas discordâncias significativas tanto nas comparações de pares de resultados (p=0,549), como na taxa de contaminação das culturas (p=0,065). O grau de concordância das técnicas foi considerado excelente (índice Kappa de 0,877), e o Ogawa-Kudoh, em relação ao Petroff modificado, apresentou 90,4% de sensibilidade, 96,6% de especificidade, 94,3% de valor preditivo positivo e 94,2% de valor preditivo negativo.

**Conclusão:**

O método de Ogawa-Kudoh revelou-se suficientemente sensível e específico para o diagnóstico da tuberculose pulmonar e, portanto, adequado para a aplicação na rotina laboratorial. Por ser mais simples, de baixo custo e com menores exigências técnicas de biossegurança e capacitação profissional, o Ogawa-Kudoh apresenta-se como alternativa para gestores e profissionais da área promoverem a ampliação da cobertura diagnóstica bacteriológica da tuberculose pulmonar.

## INTRODUCTION

The tuberculosis (TB) epidemic is larger than previously announced, although the number of deaths and its incidence have been decreasing globally. According to the World Health Organization (WHO), 10.4 million new cases were estimated worldwide in 2015, with approximately 1.4 million deaths. Brazil occupies a prominent position within this scenario; it ranks 16^th^ in absolute numbers (81,137 cases reported in 2015) and 20^th^ in incidence (41.0/100 thousand inhabitants), considering the list of the 20 countries responsible for approximately 84.0% of the world’s burden of TB.^(^ ^1^ ^)^

In order to meet the goals proposed by the Global End Tuberculosis Strategy^(^ ^2^ ^)^ report by the WHO (a 95.0% reduction in the absolute number of deaths by TB and a 90.0% drop in incidence rate by 2,035, compared with data from 2015), it is essential that the diagnosis occur in a quick and precise manner. The clinical picture and radiological modifications can provide strong signs of the disease, but the definitive diagnosis is reached by verifying the presence of the etiologic agent in respiratory samples or those coming from other sites.^(^ ^3^ ^,^ ^4^ ^)^

Culture is still considered the gold standard in the diagnosis of mycobacterium infections.^(^ ^1^ ^,^ ^3^ ^)^ If every possibility of sample contamination is excluded, a positive culture result, even in asymptomatic cases or with a normal chest X-ray, allows making diagnosis of TB. This is a method with high specificity and sensitivity, which compared to the exclusive diagnosis by bacilloscopy, can increase the bacteriologic diagnosis of the disease by up to 30.0%. Moreover, it allows further identification of the isolated mycobacterium and susceptibility tests to antimicrobial agents.^(^ ^5^ ^)^ Nevertheless, before inoculation, it is necessary to treat the samples of nonsterile sites to eliminate microorganisms of the natural and environmental microbiota, favoring the growth and isolation of the mycobacteria.^(^ ^6^ ^)^

The modified Petroff method is the standard for performing decontamination of the samples, and the Löwenstein Jensen solid culture medium is used for inoculation.

However, this is a method with many steps that requires a longer time of execution, use of a refrigerated centrifuge, and a biosafety cabinet.^(^ ^6^ ^)^ The demands and costs of a large technical infrastructure and a qualified team, associated with the budget limitations of the healthcare systems of Brazil and other emerging countries, may represent barriers for performing the test.^(^ ^7^ ^,^ ^8^ ^)^ According to data from the Information System on Reportable Diseases (SINAN - *Segundo dados do Sistema de Informação de Agravos de Notificação*), and of the Mortality Information System (SIM - Sistema de Informação de Mortalidade), between 2002 and 2009, the sputum culture was only performed in 12.9% of cases reported in the State of Minas Gerais.^(^ ^9^ ^)^

Thus, the development and/or use of simpler methodologies with lower cost and equal sensitivity during the decontamination stage of the samples for posterior inoculation would increase the possibility of performing cultures in the laboratory routines of different regions, and consequently, the diagnostic coverage of TB. In this sense, the Ogawa-Kudoh method is simplified for decontamination, lasting from 3 to 4 minutes, and dispensing the centrifugation step and use of the biosafety cabinet; therefore it is more economical.^(^ ^6^ ^)^ The Ministry of Health proposed the use of the Ogawa-Kudoh method for performing cultures, offering technical and financial support, as well as training in a few strategic capital cities or towns.^(^ ^5^ ^)^

## OBJECTIVE

To compare the performance of the Ogawa-Kudoh and modified Petroff methods in sputum samples of patients with tuberculosis, and verify if the Ogawa-Kudoh method is appropriate for the situation and profile of the disease.

## METHODS

In the *Zona da Mata* region of the state of Minas Gerais, Juiz de Fora, Brazil is considered a hub city and a strategic municipality.^(^ ^10^ ^,^ ^11^ ^)^ The study was conducted at a public tertiary care hospital, a reference in treatment of TB in this region. A total of 205 sputum samples from 166 patients with clinical suspicion or under pulmonary TB follow-up, seen between July 2014 and July 2016, were evaluated. The research protocol was approved by the Ethics in Research with Human Beings Committee of the *Fundação Hospitalar do Estado de Minas Gerais* (FHEMIG), with opinion number 816.628/2014, and CAAE #: 36661114.0.0000.5119.

Each sample was divided into two equal aliquots for simultaneous application of the modified Petroff and Ogawa-Kudoh methods, as per recommendations of the Ministry of Health.^(^ ^6^ ^)^ For each sample, two slides were prepared - one to evaluate the sputum quality (Gram staining technique) and the other to investigate alcohol-acid resistant bacilli (Ziehl-Neelsen staining technique).

To decontaminate the samples by the Ogawa-Kudoh method, a cotton swab was introduced into the most purulent portion of the sputum, and then it was submerged into a 4% solution of sodium hydroxide (NaOH) for 2 minutes. The excess of 4% NaOH was removed by compression of the swab against the flask wall and then, inoculation was performed, in duplicate, in the Ogawa-Kudoh medium (a slightly acidified medium, pH 6.4).

For decontamination by the modified Petroff method, 2mL of sputum were transferred to a Falcon-type tube. The same volume of 4% NaOH solution was added to the tube, and the mixture was homogenized and placed for 15 minutes at a temperature of 36±1°C for fluidification-decontamination. Later, 4mL of sterile distilled water were added and the neutralizing solution was dripped until change of color was visualized − from pink to amber yellow. After this stage, the resulting solution was centrifuged at 3,000 rotations per minute (rpm), during 15 minutes. The supernatant was discarded in a splash-proof recipient, while the sediment was homogenized, and 0.2mL of it was inoculated in duplicate into the Löwenstein Jensen medium.

The inoculated culture media were incubated at a temperature of 36±1°C and checked weekly. Those with growth were separated for confirmation via bacilloscopy, while the others were reincubated. After 60 days of incubation, if there was no growth of microorganisms consistent with the morphology of mycobacteria, the cultures were released as negative. Confirmed positive cultures were forwarded to *Fundação Ezequiel Dias* (FUNED), where identification was carried out.

As to statistical analyses, the parameters of sensitivity, specificity, positive predictive value, and negative predictive value of the Ogawa-Kudoh method were determined, and compared to the modified Petroff method. The McNemar test was used to compare agreement of methods, with a fixed significance level set at 0.05. To verify the agreements among data, the Kappa index was calculated, interpreted according to the Sim and Wright revision. The degree of contamination of the culture media was also assessed with both techniques. The calculations, including the descriptive statistical analysis, were made using the Statistical Package for the Social Sciences (SPSS) software, version 14.0.

## RESULTS

This study analyzed 205 samples of sputum from 166 patients in a situation of TB diagnosis or follow-up, processed simultaneously by the Ogawa-Kudoh and modified Petroff decontamination methods. The results of the cultures are detailed on table 1, using the value of p=0.549 to compare agreement of the methods, and of p=0.065 to compare the contamination rates of samples among the tests. The Kappa index was 0.877. Relative to the modified Petroff method, the Ogawa-Kudoh method displayed 90.4% sensitivity, 96.6% specificity, 94.3% positive predictive value, and 94.2% negative predictive value.

**Table 1:** Sputum cultures with decontaminated samples using the modified Petroff and Ogawa-Kudoh methods

Culture results	Modified Petroff n (%)	Ogawa- Kudoh n (%)	*p value
Isolation of mycobacteria†	76 (37,1)	71 (34,6)	0,549
Culture negative for mycobacteria	123 (60,0)	121 (59,0)	
Culture contamination	6 (2,9)	13 (6,4)	0,065

^*^ McNemar test for paired samples, with a significance level of 0.05; ^†^ identification of positive mycobacteria cultures conducted by the *Fundação Ezequiel Dias*.

## DISCUSSION

In this study, the performance of the Ogawa-Kudoh and modified Petroff methods was equivalent, both in terms of diagnostic agreement and contamination rates. Further, the Kappa index indicated a high degree of agreement between these methods. Ogawa-Kudoh showed high sensitivity, specificity, and agreement relative to the modified Petroff method, corroborating data in literature,^(^ ^8^ ^,^ ^12^ ^-^ ^17^ ^)^ and fully capable of being an important alternative for the diagnosis of TB.

Since clinical laboratories play a significant role in the follow-up of TB, the possibility of a quick diagnosis, with a simpler and cheaper execution, such as the Ogawa-Kudoh method, favors a series of activities that help in diminishing the propagation of the disease.^(^ ^7^ ^,^ ^18^ ^,^ ^19^ ^)^ Culture remains as the gold standard for diagnosis of TB, despite more advanced techniques available on the market, and the laboratories are crucial to assess quality of samples for a better performance of the test.^(^ ^3^ ^,^ ^6^ ^)^

Implementing the Ogawa-Kudoh method, especially in priority regions and/or those of difficult access, was suggested by other studies, including within the context of different healthcare services.^(^ ^12^ ^-^ ^17^ ^)^ Takao et al.,^(^ ^12^ ^)^ evaluating sputum samples of patients seen at Primary Care Units, determined that the Ogawa-Kudoh method, compared to the modified Petroff method, proved to be efficient to detect *Mycobacterium tuberculosis* and mycobacteria not belonging to the *M. tuberculosis* complex, even in samples with negative bacilloscopy. The authors found no statistical differences in contamination rates observed by both methods.

Silva et al.,^(^ ^13^ ^)^ evaluated a 8-year period of use of the Ogawa-Kudoh method in the routine of a reference laboratory in the Northeastern region of the State of Paraná, and obtained results that allowed concluding the technique is an excellent tool for early diagnosis of pulmonary TB. Some studies from other countries, such as those conducted in Venezuela, Uruguay, and Gambia, also demonstrated satisfactory results and adjustment of the Ogawa-Kudoh method to the needs and infrastructure of these different locations.^(^ ^14^ ^-^ ^16^ ^)^

In other studies, the Ogawa-Kudoh method was also compared with other forms of sample pre-treatment. Palaci et al.,^(^ ^8^ ^)^ showed 94.8% sensitivity and 99.8% specificity of the Ogawa-Kudoh method based on the cases confirmed by cultures processed with N-acetyl-L-cysteine-sodium hydroxide (NALC-NaOH) and inoculated in Löwenstein Jensen medium. Further, the culture by Ogawa-Kudoh contributed significantly to diagnosis of pulmonary TB in four different regions of Brazil (São Paulo, Espírito Santo, Rio Grande do Sul and Mato Grosso do Sul).^(^ ^8^ ^)^ Oliveira et al.,^(^ ^17^ ^)^ obtained results that allowed them to conclude that Ogawa-Kudoh could replace the method using sodium lauryl sulfate/Löwenstein Jensen with no losses in the search for cases in the National Program for Control of Tuberculosis in the State of Rondônia, MT, Brazil.

As to the medium used, it is currently sold ready for use in Brazil, and is even furnished by the Ministry of Health, which recommends its use as an attempt to decentralize the diagnosis of TB and have more lung sample cultures, favoring the diagnosis and consequently, the follow-up of the disease. Furthermore, smaller laboratories, with less sophisticated structures, are able to perform this test − which would not be possible by centrifugation.^(^ ^19^ ^)^

The Ogawa-Kudoh method is known, but not used as it could be. Some studies showed its feasibility, including in reference regions, can contribute towards its greater use. Verification of its reproducibility and efficacy in different regions, in distinct contexts of types and levels of healthcare service or population, is of paramount importance to consolidate the technique. By doing so, managers will have more evidence and support to make decisions on its implementation. There are published studies comparing the techniques, but still no conclusive discussion. For this reason, and considering the importance of TB in the context of public health worldwide, the authors deemed this article innovative.

The organization in the *Zona da Mata* region of Minas Gerais is a reference center in the treatment of TB, representing an important site of study of this disease. Additionally, these pieces of information are relevant and strategic for managers, healthcare professionals, and specialists in the subject to support their decisions on a decontamination method. The aim is to reach greater laboratory coverage of pulmonary TB, and subsidize the decision of the Ministry of Health to extend the use of this method.

## CONCLUSION

The Ogawa-Kudoh method was efficient to detect mycobacteria when compared to the modified Petroff method, and no significant discrepancies were observed when comparing test results and feasibility of the isolates (absence of contamination). The agreement between techniques was considered excellent. The findings of this study are applicable to the needs of patients in the region, and can be extrapolated to other patient populations of different regions.
